# Sentinel Lymph Node Biopsy (SLNB) for Early-Stage Head and Neck Squamous-Cell Carcinoma of the Tongue: Twenty Years of Experience at I.N.T. “G.Pascale”

**DOI:** 10.3390/cancers16061153

**Published:** 2024-03-14

**Authors:** Franco Ionna, Ettore Pavone, Corrado Aversa, Francesco Maffia, Raffaele Spinelli, Emanuele Carraturo, Giovanni Salzano, Fabio Maglitto, Marco Sarcinella, Roberta Fusco, Vincenza Granata, Secondo Lastoria, Francesco Del Prato, Maria Grazia Maglione

**Affiliations:** 1 Istituto Nazionale Tumori—IRCCS—Fondazione “G.Pascale”, 80131 Naples, Italy; f.ionna@istitutotumori.na.it (F.I.); e.pavone@istitutotumori.na.it (E.P.); c.aversa@istitutotumori.na.it (C.A.); v.granata@istitutotumori.na.it (V.G.); s.lastoria@istitutotumori.na.it (S.L.); f.delprato@istitutotumori.na.it (F.D.P.); mariagrazia.maglione@istitutotumori.na.it (M.G.M.); 2 Maxillofacial Surgery Unit, Department of Neurosciences, Reproductive and Odontostomatological Sciences, University Federico II, 80131 Naples, Italy; raffaele.spinelli.1991@gmail.com (R.S.); emanuele.c2971995@gmail.com (E.C.); giovannisalzanomd@gmail.com (G.S.); sarcinellaamarco@gmail.com (M.S.); 3 Maxillofacial Surgery Operative Unit, Department of Interdisciplinary Medicine, Aldo Moro University of Bari, 70120 Bari, Italy; fabio.maglitto@policlinico.ba.it; 4 Medical Oncology Division, Igea SpA, 80013 Naples, Italy; r.fusco@igeamedical.com

**Keywords:** head and neck cancer, oral tongue cancer, sentinel lymph node biopsy, neck metastasis, neck dissection, occult metastasis, oral tongue squamous-cell carcinoma

## Abstract

**Simple Summary:**

This study presents the experience of a single center in sentinel lymph node biopsy (SLNB) as an alternative to elective neck dissection (END) in T1/T2 cN0 oral tongue squamous-cell carcinoma (OTSCC) patients. A 20-year retrospective analysis was conducted at the Ear Nose Throat (ENT) and Maxillofacial Surgery department of the Istituto Nazionale Tumori in Naples. Between January 2002 to January 2022, 122 patients were eligible and were enrolled. Out of 122 patients, 24.6% showed positivity in SLN biopsy, with a 21.9% positivity ratio for resected nodes. Preoperative radioactive tracer injection and lymphoscintigraphy facilitated sentinel lymph node identification. This study suggests SLNB as a reliable staging tool, enabling early micrometastasis detection in clinically negative necks in patients affected by OTSCC.

**Abstract:**

Oral tongue squamous-cell carcinoma (OTSCC) is the most prevalent malignancy in the head and neck region. Lymphatic spread, particularly to cervical lymph nodes, significantly impacts 5-year survival rates, emphasizing the criticality of precise staging. Metastatic cervical lymph nodes can decrease survival rates by 50%. Yet, elective neck dissection (END) in T1–2 cN0 patients proves to be an overtreatment in around 80% of cases. To address this, sentinel lymph node biopsy (SLNB) was introduced, aiming to minimize postoperative morbidity. This study, conducted at the ENT and Maxillofacial Surgery department of the Istituto Nazionale Tumori in Naples, explores SLNB’s efficacy in early-stage oral tongue squamous-cell carcinoma (OTSCC). From January 2020 to January 2022, 122 T1/T2 cN0 HNSCC patients were enrolled. Radioactive tracers and lymphoscintigraphy identified sentinel lymph nodes, aided by a gamma probe during surgery. Results revealed 24.6% SLN biopsy positivity, with 169 SLNs resected and a 21.9% positivity ratio. The study suggests SLNB’s reliability for T1-2 cN0 OTSCC patient staging and early micrometastasis detection.

## 1. Introduction

Head and neck squamous-cell carcinoma (HNSCC) stands as the 16th most prevalent carcinoma globally [1,2,3,4]. It is defined as a malignant neoplasm affecting the oral cavity, encompassing specific subsites such as the buccal mucosa, the floor of the mouth, tongue, alveolar ridges, retromolar trigone, hard palate, and the inner part of the lips [5,6]. The tongue, particularly in oral tongue squamous-cell carcinoma (OTSCC), is the most frequently impacted anatomical region [7]. Largely inclined to metastasize through lymphatic routes, the involvement of cervical lymph nodes historically serves as a pivotal prognostic factor [8]. The presence of undetectable metastatic cervical lymph nodes via computed tomography (CT) or magnetic resonance imaging (MRI) can reach up to 33%, diminishing the 5-year survival rate by 50% [9]. Hence, the accurate staging and identification of affected lymph nodes are imperative for appropriate treatment planning and disease management.

A comprehensive exploration of cases and the detection of neck metastases necessitate clinical examination and modern imaging techniques such as ultrasound, MRI, CT, and positron emission tomography (PET) [1,10,11]. Despite the assistance provided by diagnostic technologies, as many as 20–30% of clinically N0 patients are discovered to have a metastatic involvement of cervical lymph nodes during elective neck dissection (END) [12,13]. Consequently, the surgical management of T1-T2 OTSCC with a clinically negative neck is a subject of contention [14]. Three proposed therapeutic approaches include sentinel lymph node biopsy (SLNB), elective neck dissection (END), and watchful waiting [15]. To mitigate the risk of overtreatment and minimize postoperative morbidity linked to END, the concept of sentinel lymph node biopsy was introduced for patients with oral squamous-cell carcinoma [16]. END in T1–2 cN0 patients can guarantee high oncological safety, although studies have shown that it may represent an overtreatment in up to 80% of pathologically N0 patients [7]. Thus, the incidence of postoperative complications in patients treated for elective neck dissection ranges between 20% and 40% [6]. The goal of SLNB is to identify the first lymph nodes draining the tumor area so that they can be selectively removed [17]. In the literature, the Sentinel European Node Trial (SENT) study on SLNB reported a sensitivity of 86% and a negative predictive value of 95% [18]. SLNB represents a surgical procedure with reduced morbidity when compared with END. Whenever SLNB shows neck lymph node positivity, elective neck dissection should be performed [1]. At the National Cancer Institute (Instituto Nazionale Tumori) IRCCS “G. Pascale” of Naples, SLNB was introduced in 1997, and until 2000, a feasibility study was conducted. Then, in 2002, the SLNB procedure was introduced into regular clinical practice. This study aims to describe the last twenty years of experience in sentinel lymph node biopsy in the staging and treatment of early-stage oral tongue cancer. Particular attention was paid to the evaluation of long-term postoperative follow-up, to describe the overall and disease-free survival outcomes of the chosen procedure.

## 2. Materials and Methods

A retrospective examination was conducted on patients with clinically node-negative (cN0) T1-T2 oral tongue squamous-cell carcinoma (OTSCC) who underwent sentinel lymph node biopsy (SLNB) between January 2002 and January 2022 at the Ear Nose Throat (ENT) and Maxillofacial Surgery department of the National Cancer Institute (Instituto Nazionale Tumori, INT) IRCCS “G. Pascale” of Naples. Our institution has been a recognized center for SLNB of the head and neck region since 2002. The data were obtained from the institution’s database, adhering to the Helsinki Declaration, and were approved by the local ethics board. Prior to any diagnostic or therapeutic procedure, written informed consent was obtained from all patients for the publication of their data in an anonymous form.

Inclusion criteria comprised the following:

- Histologically confirmed early-stage OTSCC (T1-T2).
- OTSCC with no clinically or radiographically detectable regional or distant metastasis at presentation (cN0-cM0), assessed through neck ultrasound, CT, and/or MRI.
- Treatment involved the excision of the primary tongue tumor and SLNB.
- Primary OTSCC that had not undergone prior treatment.
- Absence of radiotherapy or chemotherapy in the clinical history.
- No previous occurrence of cancers at other sites.
- A minimum follow-up period of 1 year.

The diagnostic process for all patients encompassed the following:

- Clinical examination.
- Routine blood sample analysis, including liver and renal function tests.
- Preoperative flexible fibropharyngoscopy.
- Neck ultrasound with Doppler.
- Head and neck CT or MRI with contrast.
- Lymphoscintigraphy to identify the sentinel lymph node.
- Histopathological examination.

A head and neck surgeon conducted the clinical evaluation for all patients, considering the tumor site, diagnostic images, and biopsy results to determine the surgical strategy. The follow-up period was scrutinized for overall survival (OS), disease-free survival (DFS), and disease-specific survival (DSS) at 1 and 5 years. To ensure an updated histological description, all OTSCCs were retrospectively classified according to the 8th American Joint Committee on Cancer (AJCC) classification. Additionally, postoperative specimens analyzed before 2019, stored for scientific purposes in dedicated laboratories, were retrospectively assessed.

### Statistical Analysis

Data were expressed in terms of median value ± standard deviation (std). The chi-square test with Yates correction was used to verify statistically significant differences in percentage values, and the Mann–Whitney test was used to verify statistically significant differences between independent continuous variables. The McNemar test was performed to verify statistically significant differences between the overall survival of the two groups. Survival estimates were calculated with the Kaplan–Meier test. A *p*-value < 0.05 was considered statistically significant. All analyses were performed using the Statistics Toolbox of Matlab R2007a (The MathWorks Inc., Natick, MA, USA).

## 3. Results

A total of 142 patients were treated for early-stage OTSCC within the chosen period. Among the treated patients, 20 were excluded for not fulfilling the inclusion criteria: 15 because they were treated before 2018 and had a DOI > 10 mm, corresponding to T3 according to the 8th AJCC classification; and 5 were excluded due to a history of radio- and/or chemotherapy. Therefore, 122 patients met the inclusion criteria and were included in the study. Patients’ characteristics are displayed in Table 1. The male/female ratio was exactly 50% (61/61), with a median age of 59 years (range: 20–85). Histopathological examination revealed 73 pT1 (59.8%) and 49 pT2 (40.2%). A statistically significant difference was observed between patients with negative and positive SLN for the T stage (*p* value = 0.0003 at Yates’ chi-square test). The G classification showed a prevalence of G2 tumors at 51.6%, followed by G3 (30.3%), and G1 (10.7%). A statistically significant difference was observed between patients with negative and positive SLN for grading (*p* value = 0.006 at Yates’ chi-square test). SLN detection was accomplished preoperatively in all patients (100%) by means of lymphoscintigraphy and SPECT/CT. Intraoperatively, the gamma probe detection rate was successful in all cases (100%). Thirty patients out of one hundred and twenty-two (24.6%) had positivity in the SLN biopsy. In total, 169 SLNs were resected from the 122 patients, with a positivity ratio of 21.9% (37/155). More than half of positive SLNs were found in the II level (56.1%, 20/37), followed by the III level with 27% (10/37). The subsequent positivity rate among END-treated patients was 41.3% (12/29).

**Figure 1:**
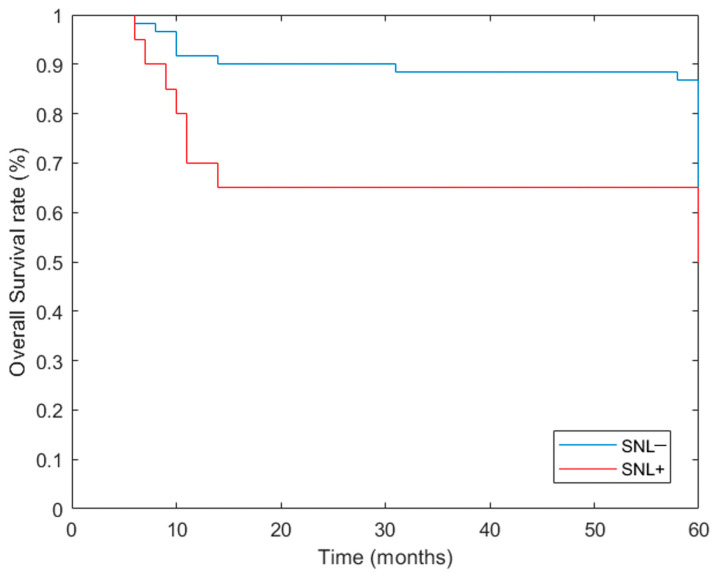
Overall survival rate of patients with positive (red line) and negative SNL (blue line).

At 5 years, the general population OS was 81.5% (66/81), with 41 patients lost at follow-up (10 in the SLNB+ group and 31 in the SLNB− group). Between the two groups, the higher 5-year OS was observed in the SLNB− group with 86.9% (53/61), while in the SLNB+ group, the 5-year OS was 65.0% (13/20). The 5-year DFS in the general population was 81.5% (66/81). The percentage was higher in the SLNB− group with 88.5% (54/61), while in the SLNB+ group, the percentage was 60.0% (12/20). At 5 years, the general DSS was 91.4% (74/81). A higher 5-year DSS was observed in the SLNB− group (95.1%, 58/61), while in the SLNB+ group, the 5-year DSS was 80.0% (16/20). 5 years survival rates are displayed in Table 2 and Figure 1.

## 4. Discussion

Oral squamous-cell carcinoma (OSCC) is a prevalent and aggressive form of cancer that originates in the epithelial cells lining the oral cavity [1,2]. Accounting for the majority of oral malignancies, OSCC poses a significant global health burden with diverse etiological factors, clinical presentations, and therapeutic challenges [2]. OSCC is one of the most common malignancies of the head and neck region, affecting various anatomical sites within the oral cavity, including the lips, tongue, the floor of the mouth, gingiva, and palate [2,3]. While the exact cause of OSCC is multifactorial, tobacco use, both smoking and smokeless forms, and excessive alcohol consumption are well-established risk factors. Human papillomavirus (HPV) infection, particularly with high-risk subtypes such as HPV-16, has also been implicated in the rising incidence of OSCC, particularly in younger individuals. Additionally, chronic exposure to ultraviolet (UV) radiation, poor oral hygiene, genetic predisposition, and dietary factors contribute to the complex etiology of this malignancy [4,5]. The clinical presentation of OSCC varies depending on the site of origin. Common early symptoms include persistent oral ulcers, non-healing sores, or white or red patches (leukoplakia or erythroplakia) in the oral mucosa [5]. As the disease progresses, individuals may experience pain, difficulty in chewing or swallowing, changes in voice, and the presence of palpable neck masses, indicating regional metastasis. Early detection is crucial for optimal outcomes, emphasizing the significance of routine oral examinations, especially for individuals with known risk factors. Diagnosing OSCC involves a comprehensive approach, combining clinical evaluation, imaging studies, and confirmatory biopsy. Biopsy specimens are obtained through techniques such as incisional or excisional biopsy, allowing for histopathological examination to confirm the presence of squamous-cell carcinoma. Additionally, imaging modalities like computed tomography (CT), magnetic resonance imaging (MRI), and positron emission tomography (PET) scans aid in staging the disease, assessing the extent of local invasion, and detecting regional and distant metastases [3,4,5].

The 5th edition of the World Health Organization (WHO) classification of head and neck tumors provides a comprehensive framework for categorizing oral squamous-cell carcinoma (OSCC) subtypes based on histopathological features. OSCC, the most common malignancy of the oral cavity, exhibits considerable heterogeneity. The WHO classification divides OSCC into several subtypes, considering factors such as tissue differentiation, growth patterns, and molecular characteristics. Notable subtypes include conventional squamous-cell carcinoma, verrucous carcinoma, papillary squamous-cell carcinoma, and basaloid squamous-cell carcinoma. Each subtype is characterized by distinctive morphological features and clinical behavior.

Conventional squamous-cell carcinoma, the predominant subtype, is further classified into well, moderately, and poorly differentiated variants. Verrucous carcinoma is distinguished by its exophytic, warty appearance, often exhibiting a less aggressive course. Papillary squamous-cell carcinoma is typified by finger-like projections of neoplastic epithelium, while basaloid squamous-cell carcinoma is associated with basal cell differentiation and tends to demonstrate increased aggressiveness [6]. The staging of OSCC is typically based on the TNM classification system, considering the tumor size, nodal involvement, and the presence of distant metastasis. Staging guides treatment planning and provides valuable prognostic information. Early-stage OSCC confined to the primary site may have a more favorable prognosis, while advanced-stage disease with extensive invasion and metastasis poses greater therapeutic challenges and carries a poorer prognosis [6].

SLNB has become an important tool for the treatment of OTSCC [7]. SLNB allows for the identification of the first lymph node that drains the tumor area, and its biopsy can provide valuable information regarding the presence or absence of occult neck metastasis [19,20,21,22]. In recent years, several studies have investigated the diagnostic and prognostic value of SLNB in OTSCC [23,24,25,26]. Civantos et al. conducted a multi-institutional study on the use of SLNB in patients with OTSCC. The study included 140 patients who underwent SLNB, and the authors found that the procedure was an effective method for identifying lymph node metastases, with a sensitivity of 96% and a negative predictive value of 96%. The authors also found that SLNB had a low false negative rate. indicating that it could reliably identify patients who did not have lymph node metastases. The study suggested that SLNB could be a reliable tool in the planning of surgical treatment for OTSCC [24,27,28,29]. Similarly, Liu M. et al. conducted a meta-analysis on the use of SLNB in the treatment of early OSCC. The analysis included 66 studies with a total of 3566 patients, and the authors found that SLNB had a high accuracy rate in identifying lymph node metastases, with a pooled sensitivity of 87% and a negative predictive value of 94%. The authors also noted that SLNB had a low complication rate and was well tolerated by patients [23,30,31]. Another meta-analysis by Yang et al. showed that SLNB had a pooled sensitivity of 82% and a specificity of 98% in cT1/T2 N0 tongue SCC. These studies provided further evidence for the effectiveness of SLNB in the treatment of OSCC and suggest that it could be a safe and reliable alternative to a more invasive elective dissection [25].

A study conducted by Hingsammer et al. aimed to evaluate the outcomes of sentinel lymph node biopsy in early-stage tongue cancer patients and its impact on OS and DFS. The researchers conducted a 14-year single-center study, analyzing the data of tongue cancer patients who underwent SLNB. At 5 years, the OS was 80% and the DSS was 95%. Tumor recurrence or progression was observed in two patients (5%), revealing a 5-year DFS of 95%. In the patients who had positive sentinel lymph nodes, further lymph node dissection was performed, leading to a more accurate staging and subsequent treatment. In terms of overall survival, patients with negative sentinel lymph nodes had a significantly higher survival rate compared to those with positive nodes. Regarding disease-free survival, the study demonstrated that patients with negative sentinel lymph nodes had a lower risk of tumor recurrence or metastasis. SLNB provided valuable information for accurate staging and subsequent treatment decisions, potentially leading to improved disease control and long-term outcomes. The results obtained in terms of overall survival and disease-free survival support the use of SLNB as a beneficial approach in the management of early-stage tongue cancer patients [7].

Salzano et al. found that a positive SLNB was associated with a worse prognosis in early-stage OTSCC. The authors also identified a correlation between pre-treatment inflammatory biomarkers, the depth of invasion, and the worst pattern of invasion with a positive SLNB. These findings suggest that SLNB can be associated with valuable prognostic information for patients with early OTSCC, providing decisional support in the surgical treatment [26]. Park et al. reported that SLNB had comparable long-term oncologic outcomes to END in clinically node-negative tongue SCC. The authors found no significant difference in locoregional recurrence, distant metastasis, disease-specific survival, or overall survival between the two groups [32]. Fan et al. also reported that SLNB had a similar recurrence rate to END in cT1-2N0 tongue SCC [27].

In our study, 24.6% of the cohort showed sentinel lymph node positivity, and out of the total of 169 sentinel lymph nodes (169), the positivity rate was 21.9%. Of the 37 positive sentinel lymph nodes, 56.1% were found in level II and 27% in level III. At 1 year after surgery, the OS of the study population was 66.7% for the cohort found positive at sentinel lymph node biopsy (SNLB+) and 93.2% for the cohort found negative at sentinel lymph node biopsy (SNLB−). This trend was confirmed at 5 years, as an OS value of 86.7% was recorded for the SNLB− cohort and 65% for the SNLB+ cohort. A similar result was observed for the DFS value, whereby at 1 year after surgery, the DFS value for the cohort with negative sentinel lymph node biopsy stood at 90.7%, while the cohort with positive sentinel lymph node biopsy stood at 66.7%. At 5 years, the trend for this value also remained similar, as the SNLB− cohort showed values of 88.5% and the SNLB+ cohort of 60%. Regarding the DSS value, in the SNLB− cohort, it was 97.7% and in the SNLB+ cohort, it was 80% at 1 year after biopsy, while at 5 years, values of 95.1% were recorded for the SNLB− cohort and 80% for the SNLB+ cohort. 

The relapse rate at 1 year was 9.3% in the SLNB− group and 33.3% in the SLNB+ group, and at 5 years, it was 11.7% in the SNLB− cohort and 40% in the SNLB+ cohort. All these values showed statistical significance, except in the case of the differences between the 5-year overall survival values. Our results were in line with the literature: a review by Cramer et al. showed similar values for OS. In their review, the 3-year OS was 82.0% after SLNB and 77.5% after END [33]. 

Rigual N. et al. reported similar outcomes. In the outcome findings, it was observed that 2 individuals out of the cohort of 33 patients with negative sentinel lymph node biopsy (SNB) results experienced regional recurrence. The sensitivity and negative predictive value for neck staging solely with SNB were determined to be 71% and 94%, respectively. Regarding disease-specific survival rates, patients with positive SNB results exhibited an 80% rate, while those with negative SNB results demonstrated a higher rate at 91% [34]. Flach et al. reported the following results: the DFS, OS, and DSS of SLN-negative patients were 72.0%, 92.7%, and 97.4%, and for SLN-positive patients, these values were 73.7%, 79.7%, and 85.0%, respectively. Neck control rate was 97% in SLN-negative and 95% in SLN-positive patients [35]. 

The findings from the Sentinel European Node Trial (SENT) in 2015 revealed that 23% (94 out of 415) of patients had positive sentinel lymph node biopsy (SLNB) results. Among the patients, 14% (15 out of 109) experienced false-negative results, and 8 of them were subsequently treated with salvage therapy. Recurrence following a positive sentinel lymph node biopsy (SNLB) and subsequent neck dissection occurred in 22 patients, with 73% (16 out of 22) manifesting in the neck, and only 6 patients underwent successful rescue. Minor complications were reported in 3% of cases following SNLB. The disease-specific survival rate was reported to be 94% [18]. The results according to the multicenter phase II study by Miura et al. for the overall survival were as follows: the 3-year overall survival (OS) and disease-free survival were 89.5% and 82.5%, respectively, for the whole populations [36].

Several studies have investigated the use of SLNB with different tracers in OSCC. Doll et al. reported that SLNB using the receptor-targeted radiotracer 99mTc-Tilmanocept had a detection rate of 94% in early-stage OSCC [37]. Similarly, Huang et al. found that trans-lymphatic contrast-enhanced ultrasound with SLNB had a detection rate of 97.4% in early-stage tongue SCC. These findings suggest that the use of different tracers with SLNB can improve detection rates and accuracy in OSCC [38]. 

A technical improvement of the SLNB technique is using indocyanine green fluorescence imaging (ICG). Bredell et al. conducted a preliminary study on the application of ICG fluorescence imaging in SLNB for oropharyngeal cancer. The authors highlighted the potential increase in accuracy in lymph node mapping [39]. In a study by Al-Dam et al. (2018), the sensitivity and specificity of SLNB using ICG fluorescence imaging were evaluated in patients with a higher concentration of ICG than was used in the Bredell study (0.5 mg/kg body weight in 2 mL versus 10 mg/kg in 1 mL solution). In the Al-Dam et al. study, the first SLN was identified after an average of 8.1 min (range: 1–22 min), while in Bredell’s study, the time between injection and imaging ICG was 2–30 min (Bredell, 2010) [39,40]. The sensitivity of ICG mapping in this study was not very high, only 50%, but the incidence of false negatives and the reduced population (20) influenced this outcome. The positive predictive value was 100% and the negative predictive value was 75% [40,41,42]. All these studies collectively suggest that ICG fluorescence imaging has the potential to enhance the accuracy and precision of SLNB in head and neck oncology, notwithstanding the differences among the reported centers [39,40,41,42,43]. As firstly described by Van den Brekel et al. (1992), ultrasound-guided fine-needle aspiration cytology (FNAC) has already emerged as a valuable diagnostic tool in the assessment of neck lymphatic metastasis in oral squamous-cell carcinoma, and represents an effective and low-cost method for assess the regional spread of cancer [44]. Ultrasound-guided fine-needle aspiration cytology (FNAC) and sentinel node biopsy (SNB) are both diagnostic tools employed in the assessment of neck lymphatic metastasis in oral squamous-cell carcinoma (OSCC), yet they differ in their approaches and applications [44,45]. Ultrasound-guided FNAC utilizes real-time ultrasound imaging to target suspicious lymph nodes and obtain cytological samples through a fine needle, allowing for a direct assessment of cellular characteristics. This technique is particularly effective for confirming the presence of metastatic cells and determining the extent of lymph node involvement.

On the other hand, sentinel node biopsy involves the identification and removal of the first lymph node(s) that receive drainage from the tumor site. While sentinel node biopsy provides valuable information about the status of the primary lymphatic drainage, it may not offer as precise a characterization of individual lymph nodes as ultrasound-guided FNAC [46,47].

The choice between these diagnostic tools often depends on factors such as the stage of the disease, the location of the primary tumor, and the specific clinical scenario. Ultrasound-guided FNAC is advantageous for obtaining direct cytological evidence from suspicious lymph nodes, offering a more detailed analysis of metastatic involvement. Sentinel node biopsy, on the other hand, provides information about the primary drainage pathways and can guide decisions on the extent of neck dissection. In summary, both ultrasound-guided FNAC and sentinel node biopsy contribute valuable insights into the assessment of neck lymphatic metastasis in OSCC, with each method offering distinct advantages that may be tailored to the individual patient’s diagnostic and treatment needs. The selection of the most appropriate technique often involves a comprehensive evaluation of the specific clinical context and requirements for accurate staging and treatment planning.

## 5. Conclusions

In conclusion, sentinel lymph node biopsy stands out as a reliable and valuable tool in the management of early oral tongue squamous-cell carcinoma. This practice not only exhibits a high detection rate but also furnishes essential prognostic information for patients. Given the potential for sparing numerous patients from unnecessary invasive treatments post-SLNB nodal staging, we support the incorporation of this procedure into clinical practice. A vigilant follow-up protocol, along with a potential salvage management plan, becomes imperative for achieving excellent long-term outcomes. Nevertheless, a negative SLNB not only could eliminate the need for neck dissection but also mitigate the risk of further neck surgery-related complications for the patient.

In summary, our perspective aligns with regarding SLNB as both a reliable staging method and a suitable treatment alternative for patients in the early stages of tongue OSCC with initial clinical N0 staging.

## Figures and Tables

**Table 1:** Patients’ characteristics include demographic data, T classification, G classification, and SLNB location, with statistical analysis.

Characteristic	Overall		SLN− Patients		SLN+ Patients		*p* Value at Yates’ Chi-Square Test or Mann–Whitney Test
	N°	%	N°	%	N°	%	
Patients	122	100.0%	92	75.4%	30	24.6%	
Sex							
Male	61	50.0%	51	55.4%	10	33.4%	
Female	61	50.0%	41	44.6%	20	66.6%	0.06
Age	59	20–85	60	20–85	57	31–79	0.23
T Classification							
T1	73	59.8%	64	69.6%	9	30.0%	
T2	49	40.2%	28	30.4%	21	70.0%	0.0003
G Classification							
G1	13	10.7%	11	12.0%	2	6.6%	
G2	63	51.6%	55	59.8%	8	26.7%	
G2–G3	9	7.4%	6	6.5%	3	10.0%	
G3	37	30.3%	20	21.7%	17	56.7%	0.006
Location of SLN							
Level I	12	7.1%	12	9.1%	0	0.0%	
Level II	94	55.6%	74	56.1%	20	54.1%	
Level III	52	30.8%	42	31.8%	10	27.0%	
Level IV	5	3.0%	4	3.0%	1	2.7%	
Level V	6	3.5%	0	0.0%	6	16.2%	
Total	169	100.0%	132	78.1%	37	21.9%	0.0006

**Table 2:** Survival rate for two groups at 5 years. OS: overall survival; DFS: disease-free survival; DSS: disease-specific survival. Although a difference was observed between the overall survival rate at 5 years for patients with negative and positive SNL, this difference was not statistically significant (*p*-value = 0.06 at Yates’ chi-square test). The relapse rate at 5 years was 11.7% (7/61) in the SLNB− group and 40.0% (8/20) in the SLNB+ group; the difference was statistically significant, with a *p*-value = 0.01 at Yates’ chi-square test.

5 Years	OS		DFS		DSS		Relapse	
	N°	%	N°	%	N°	%	N°	%
SLN−	53/61	86.89%	54/61	88.52%	58/61	95.08%	7/61	11.66%
SLN+	13/20	65.00%	12/20	60.00%	16/20	80.00%	8/20	40.00%
*p* value at Yates’ chi-square test	0.06		0.11		0.1		0.01	

## Data Availability

Data are contained within the article.
