# Correction: Ionna et al. Sentinel Lymph Node Biopsy (SLNB) for Early-Stage Head and Neck Squamous-Cell Carcinoma of the Tongue: Twenty Years of Experience at I.N.T. “G.Pascale”. *Cancers* 2024, *16*, 1153

**DOI:** 10.3390/cancers16142551

**Published:** 2024-07-16

**Authors:** Franco Ionna, Ettore Pavone, Corrado Aversa, Francesco Maffia, Raffaele Spinelli, Emanuele Carraturo, Giovanni Salzano, Fabio Maglitto, Marco Sarcinella, Roberta Fusco, Vincenza Granata, Secondo Lastoria, Francesco Del Prato, Maria Grazia Maglione

**Affiliations:** 1 Istituto Nazionale Tumori—IRCCS—Fondazione “G.Pascale”, 80131 Naples, Italy; f.ionna@istitutotumori.na.it (F.I.); e.pavone@istitutotumori.na.it (E.P.); c.aversa@istitutotumori.na.it (C.A.); v.granata@istitutotumori.na.it (V.G.); s.lastoria@istitutotumori.na.it (S.L.); f.delprato@istitutotumori.na.it (F.D.P.); mariagrazia.maglione@istitutotumori.na.it (M.G.M.); 2 Maxillofacial Surgery Unit, Department of Neurosciences, Reproductive and Odontostomatological Sciences, University Federico II, 80131 Naples, Italy; raffaele.spinelli.1991@gmail.com (R.S.); emanuele.c2971995@gmail.com (E.C.); giovannisalzanomd@gmail.com (G.S.); sarcinellaamarco@gmail.com (M.S.); 3 Maxillofacial Surgery Operative Unit, Department of Interdisciplinary Medicine, Aldo Moro University of Bari, 70120 Bari, Italy; fabio.maglitto@policlinico.ba.it; 4 Medical Oncology Division, Igea SpA, 80013 Naples, Italy; r.fusco@igeamedical.com

## Error in Affiliation

In the published publication [1], there was an error regarding the affiliation for “Roberta Fusco”. The previous affiliation was “Istituto Nazionale Tumori—IRCCS—Fondazione “G.Pascale”, 80131 Naples, Italy”, which now should be “Medical Oncology Division, Igea SpA, 80013 Naples, Italy”, becoming the new affiliation number 4. The authors apologize for any inconvenience caused and state that the scientific conclusions are unaffected. This correction was approved by the Academic Editor. The original publication has also been updated.
